# Exploiting the *Campylobacter jejuni *protein glycosylation system for glycoengineering vaccines and diagnostic tools directed against brucellosis

**DOI:** 10.1186/1475-2859-11-13

**Published:** 2012-01-25

**Authors:** Jeremy A Iwashkiw, Messele A Fentabil, Amirreza Faridmoayer, Dominic C Mills, Mark Peppler, Cecilia Czibener, Andres E Ciocchini, Diego J Comerci, Juan E Ugalde, Mario F Feldman

**Affiliations:** 1 Alberta Glycomics Centre, Department of Biological Sciences, University of Alberta, Edmonton, AB, TG6 2E9, Canada; 2 Alberta Glycomics Centre, Department of Chemistry, University of Alberta, Edmonton, AB, T6G 2G2, Canada; 3 Department of Medical Microbiology and Immunology, University of Alberta, Edmonton, AB, T6G 2H7, Canada; 4 Instituto de Investigaciones Biotecnológicas, "Dr. Rodolfo A. Ugalde", IIB-INTECH, CONICET, Universidad Nacional de San Martín, Av. Gral. Paz 5445, PREDIO INTI, Edificio 24 (1650), Buenos Aires, Argentina; 5 Pathogen Molecular Biology Unit, London School of Hygiene & Tropical Medicine, Keppel Street, London, WC1E 7HT, UK; 6 GlycoVaxyn AG, Grabenstrasse 3, 8952 Schlieren, Switzerland; 7 Department of Biological Sciences, CW 405, Biological Sciences Bldg, University of Alberta, Edmonton, T6G 2E9, Canada

**Keywords:** Brucellosis diagnostics, glycoengineering, *Yersinia enterocolitica *O9, N-linked protein glycosylation

## Abstract

**Background:**

Immune responses directed towards surface polysaccharides conjugated to proteins are effective in preventing colonization and infection of bacterial pathogens. Presently, the production of these conjugate vaccines requires intricate synthetic chemistry for obtaining, activating, and attaching the polysaccharides to protein carriers. Glycoproteins generated by engineering bacterial glycosylation machineries have been proposed to be a viable alternative to traditional conjugation methods.

**Results:**

In this work we expressed the *C. jejuni *oligosaccharyltansferase (OTase) PglB, responsible for *N*-linked protein glycosylation together with a suitable acceptor protein (AcrA) in *Yersinia enterocolitica *O9 cells. MS analysis of the acceptor protein demonstrated the transfer of a polymer of N-formylperosamine to AcrA *in vivo*. Because *Y. enterocolitica *O9 and *Brucella abortus *share an identical O polysaccharide structure, we explored the application of the resulting glycoprotein in vaccinology and diagnostics of brucellosis, one of the most common zoonotic diseases with over half a million new cases annually. Injection of the glycoprotein into mice generated an IgG response that recognized the O antigen of *Brucella*, although this response was not protective against a challenge with a virulent *B. abortus *strain. The recombinant glycoprotein coated onto magnetic beads was efficient in differentiating between naïve and infected bovine sera.

**Conclusion:**

Bacterial engineered glycoproteins show promising applications for the development on an array of diagnostics and immunoprotective opportunities in the future.

## Background

*Brucella *sp., the causative agents of brucellosis, are Gram-negative, facultative intracellular α-proteobacteria [1-3]. Three *Brucella *species, *B. abortus*, *B. melitensis*, and *B. suis *are the common species that cause human brucellosis. They can also infect domestic livestock, causing miscarriages and sterility leading to significant economic loss [4,5]. Brucellosis is the most common bacterial zoonosis with over half a million new cases annually and high levels of abortions in cattle in developing countries [6,7]. In addition, *Brucella *sp. are considered highly effective biological weapons [1]. *B. abortus *is the causative agent for brucellosis in cattle and the second most common cause of human infections [8]. The current commercially available vaccines against *B. abortus *are attenuated strains, which are effective in livestock, but retain virulence to humans [9]. Due to this and other disadvantages, such as the impossibility to discriminate between infected and vaccinated animals during immune-screening procedures, new vaccines against brucellosis are required. Among several promising vaccine candidates is a live attenuated strain lacking the phosphoglucomutase gene (*Δpgm*), which is unable to assemble the O polysaccharide [10].

Immune responses directed towards surface polysaccharides are effective in preventing colonization and infection against several bacterial pathogens [11]. However, to generate long-term protection in children, the polysaccharides must be covalently attached to an appropriate protein carrier [11,12]. The efficacy of conjugating bacterial polysaccharides to proteins is best exemplified by the *Haemophilus influenzae *type b conjugate vaccine, which has virtually eradicated the infections caused by this organism in most parts of the world [11]. Indeed, glycoconjugate vaccines have also been used for the prevention and treatment of a diverse array of bacterial, viral, protozoan, parasitic, and cancerous diseases [11]. Presently, the production of these conjugate vaccines requires intricate synthetic chemistry for obtaining, activating, and attaching the polysaccharides to protein carriers [11]. The polysaccharides are either obtained from the target pathogen, or by laborious synthesis. Extraction of the polysaccharides from pathogenic organisms usually requires large cultures, which constitutes a major health hazard [13]. Furthermore, when purifying O antigens, chemical removal of the endotoxin is required to prevent fever [11]. In most cases, bacterial polysaccharides are too complex to be synthesized efficiently by chemical methods, which make this process economically unfavorable [13]. In the final stage of conjugation, chemical attachment of the carbohydrate to the protein often results in large and heterogeneous conjugates. In addition, a considerable amount of toxic waste is generated during the conjugation process [13]. For these reasons, production of conjugate vaccines using conventional procedures is complex and the costs are prohibitive for global vaccination programs.

The O antigen of *B. abortus *and *B. suis *is a homopolymer of N-formylperosamine [14,15]. Only a few studies evaluating the suitability of conjugate vaccines against *Brucella *have been published. A conjugate vaccine obtained by covalently coupling the O-polysaccharide obtained from *B. melitensis *to bovine serum albumin (BSA) induced antibodies and was protective in mice [16]. Nevertheless, because *Brucella *sp. requires class III biosafety facilities, production of glycoconjugates containing *Brucella *glycans in its native host is challenging and possibly unsafe. It has recently been established that conjugates containing polysaccharide from pathogenic bacteria can be produced in *E. coli *by exploitation of bacterial glycosylation systems. Bacterial oligosaccharyltransferases (OTases) are enzymes capable of transferring glycan chains, including polysaccharides, from lipid carriers to proteins, in a process called *en bloc *protein glycosylation [17]. OTases involved in both, *N*- and *O*-glycosylation have been characterized in bacteria [17,18]. The most thoroughly studied bacterial glycosylation system is the *N*-glycosylation machinery of *Campylobacter jejuni *[17,19-21]. *N-*glycosylation is initiated by a specialized glycosyltransferase that attaches a nucleotide-activated monosaccharide-1P to an undecaprenolphosphate (Und-P) lipid carrier on the inner face of the inner membrane. A series of glycosyltransferases subsequently attach additional monosaccharides to the first sugar residue on Und-PP. When the carbohydrate structure is completed, the Und-PP linked glycan is flipped to the periplasmic face, where the *N-*OTase PglB transfers the carbohydrate to protein acceptors with a consensus sequence of D/E-Y-**N**-X-S/T (Y, X ≠ P) [22,23]. Previous work has demonstrated that *C. jejuni *PglB can transfer an array of glycans, including O-antigens, from the lipid donors to carrier proteins [21]. Due to their versatility, bacterial glycosylation systems can be seen as toolboxes for engineering novel glycoconjugates. Conjugates produced by this method may constitute a new generation of vaccines, circumventing most of the disadvantages of the conventional chemical methods, significantly reducing costs, and improving the reproducibility of the product obtained. In this work, we exploited the *C. jejuni *N-glycosylation machinery to engineer *N*-linked glycoproteins and tested their possible applications in vaccinology. We also demonstrated that these glycoproteins have promising applications for the diagnosis of brucellosis.

## Results

### Cross reactivity between *Brucella *and *Y. enterocolitica *O:9

**Figure 1:**
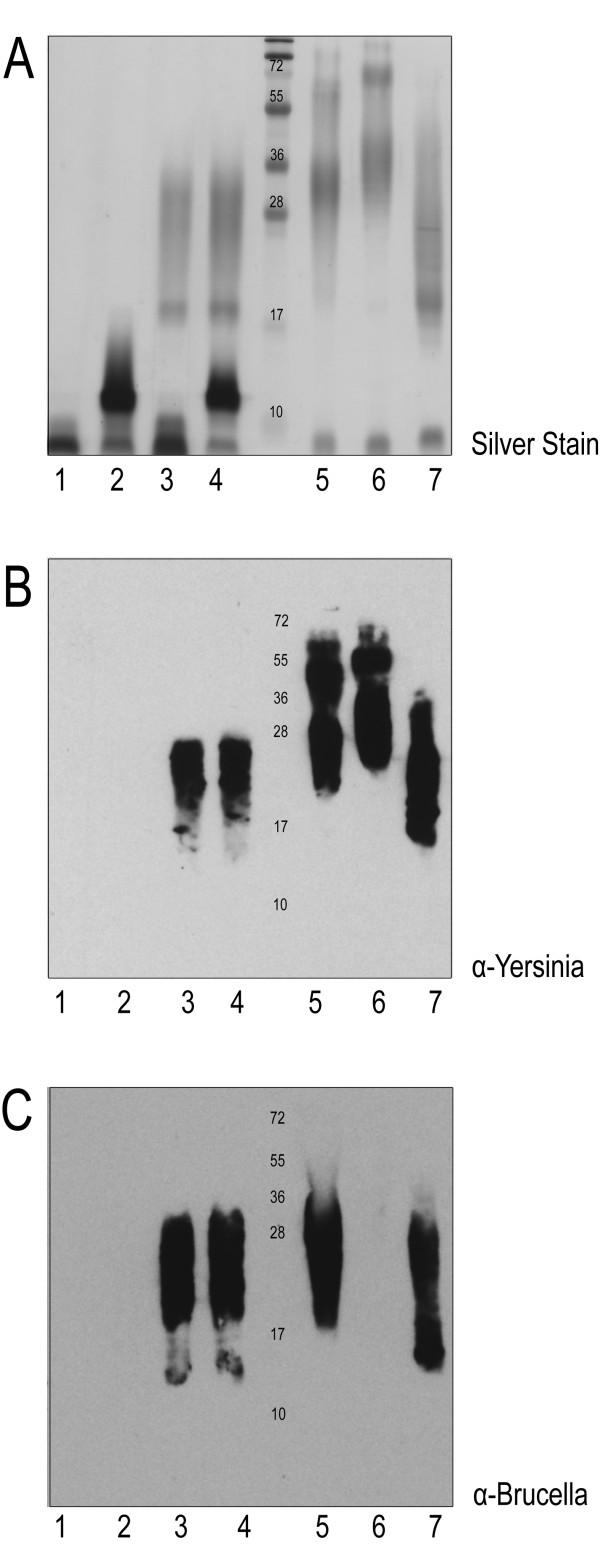
**Cross reactivity between *Y. enterocolitica *O:9 and *Brucella *spp. LPS samples**. (0.2 OD/sample loaded) on 15% SDS-PAGE: A) LPS silver stain analysis of samples of *Y. enterocolitica *1) OC-/HP-, 2)HP-, 3) OC-, 4) WT; *Brucella *5) *B. abortus*, 6) *B. melintensis*, and 7) *B. suis*. B) Immunoblot against the same samples with monoclonal α-*Yersinia *(Yst 9-2). C) Immunoblot of the same samples using monoclonal α-*Brucella *(M84). Cross reactivity between the two different genus' LPS is observed by both monoclonal antibodies reacting against the higher molecular weight homopolymeric N-formylperosamine polysaccharide.

The *B. abortus *and *B. suis *O antigens were previously characterized by genomic analysis, NMR, and serological assays and appear to be identical to that of *Yersinia enterocolitica *O:9 (Ye O:9) [15,24,25]. Ye O:9 is a Class II biosafety hazard organism and is easily manipulated and cultured, making it a suitable host for the production of the glycoconjugate protein with the N-formylperosamine homopolymer, which we hypothesize could cross-protect against brucellosis [26]. In some *Y. enterocolitica *strains, an additional "outer core" (OC) consisting of a shorter glycan chain is assembled onto the Und-PP carrier and subsequently ligated to lipid A. To confirm cross reactivity of the Ye O:9 (Table 1) and the *B. abortus *O antigens, LPS of both species were analyzed by SDS-PAGE and immunoblot (Figure 1). Our analysis included the wild-type Ye O:9 strain, plus three derivatives lacking the OC, the O antigen, or both glycan structures. LPS samples from of the Ye O:9 strains exhibited a different electrophoretic pattern according to the mutation carried by each strain. The double mutant strain only displayed a band corresponding to lipid A core (lane 1). The O antigen deficient strain exhibited a unique band that migrated slower than the lipid A core, as expected for the presence of the low molecular weight OC structure attached to the lipid A (lane 2). The OC minus strain only produced the high molecular weight homopolymer (lane 3), and the WT strain produced both glycan structures (lane 4; Figure 1A). Analysis of the LPS extracts using monoclonal antibodies (Figure 1B, C) demonstrated that only the high molecular weight carbohydrate of Ye O:9 was reactive towards the Yst9-2 (anti-Ye O:9 antigen) monoclonal antibody (mAb; Figure 1B, lanes 3 and 4). The Yst9-2 mAb antibody also recognized the O antigen of *B abortus*, *B. melitensis *and *B. suis*, confirming the cross reactivity of the LPS between the species (Figure 1B, Lanes 5-7). The M84 mAb directed against *B. abortus *O antigen also reacted with the Ye O:9 polysaccharide (Figure 1C, lanes 3 and 4). Interestingly, *B. melitensis *LPS did not react towards the M84 antibody (Figure 1C, lane 6). This result was not totally unexpected because although the *B. abortus *and *B. melitensis *polysaccharides have a similar composition, there are structural differences between their O antigens [27]. These results confirmed that the O antigens of Ye 0:9 and *B. abortus *have a similar structure, and suggested that a conjugate carrying the Ye O:9 antigen could mount an immune response that may be cross-protective against *B. abortus*.

**Table 1:** Strains and Plasmids used in this study

Strain/Plasmid	Description	Source
pMAF10	HA-tagged PglB_Cj _cloned in pMLBAD, Tmp^R^	(11)

pMH5	Soluble periplasmic hexa-His-tagged AcrA under control of Tet promoter, in pACYC, Cm^R^	(11)

YeO9-c-OC (OC-)	Δ(*wzx-wbcL*)::KmGB; OC negative; derivative of Ruokola/71-c	(35)

YeO9-OC-R (OC-/HP-)	Phage ∅R1-37-resistant spontaneous OC-negative derivative of YeO9-R1	(35)

YeO9-R1 (HP-)	Δ*per*::KmGB; rough (O-antigen negative); Km^r ^derivative of Ruokola/71	(32,35)

Ruokola/71-c (WT)	Spontaneous virulence plasmid-cured derivative of Ruokola/71	(32)

### Purification of glycosylated AcrA from *Y. enterocolitica *O:9 strains

**Figure 2:**
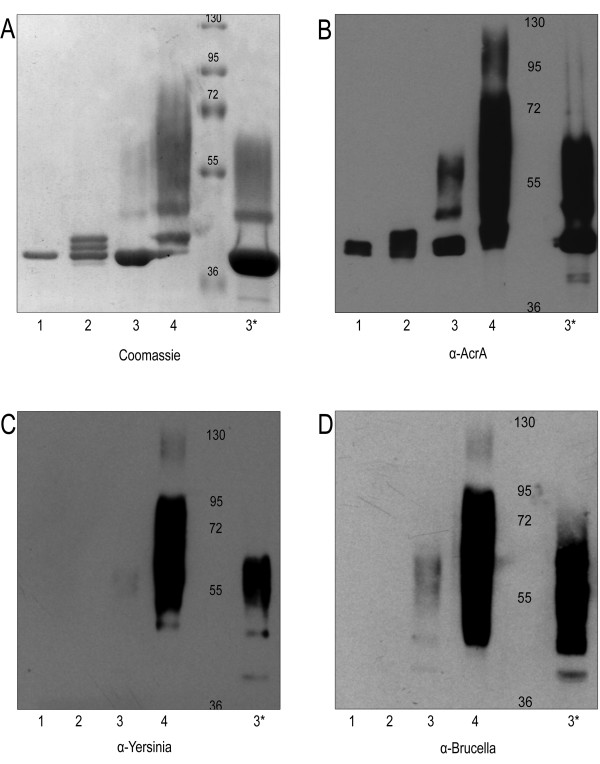
**Proteins carrying *Y. enterocolitica *O:9 O-antigens are immunoreactive against α-*Yersinia *and α-*Brucella *monoclonal antibodies**. Each strain was transformed with pMAF10 (*pglB*_Cj_) and pMH5 (*acrA*), and glycosylated AcrA was purified from 1 L of culture using Ni^2+ ^affinity chromatography. After purification, samples were loaded onto a 10% SDS-PAGE gel and analyzed by: A) Coomassie brilliant blue (5 μg/sample), immunoblot (2 μg/sample) using B) α-AcrA, C) α-Ye O:9 (Yst9-2) mAb, or D) α-*Brucella *O antigen M84 mAb. Samples were purified from the following strains: 1) OC-/HP-, 2) HP-, 3) OC-, 4) WT, and 3*) 10 × loaded volume of 3 (OC-).

In earlier work, *N*-glycosylated AcrA was synthesized in *E. coli *by co-expression of *C. jejuni *PglB and AcrA with an appropriate carbohydrate structure [22]. In order to determine if we could transfer the Ye 0:9 carbohydrate structures to AcrA in Ye O:9, we transformed each of the strains with pMAF10, expressing PglB under an arabinose-inducible promoter, and pMH5, expressing a 6-His-tagged version of AcrA. Cultures of each transformed strain were grown and induced as required, and AcrA was purified from periplasmic extracts by affinity chromatography and analyzed by SDS-PAGE (Figure 2). The single band visualized by Coomassie stain (Figure 2A) suggested that AcrA was unglycosylated in the double mutant strain (lane 1), while the two glycosylation sites of AcrA were modified with OC in the O antigen mutant strain producing an additional two bands (lane 2). The large molecular weight O antigen was transferred to AcrA in the OC mutant strain (lane 3, and 3*), and both glycan structures were bound to AcrA in the Ye O:9 WT (lane 4). Additionally, the purified AcrA samples were analyzed by immunoblot using α-AcrA antibodies (Figure 2B). The different pattern observed in each sample confirmed that different glycans were attached in each strain. Interestingly, unglycosylated AcrA appeared as a double band (lane 1). The OC mutant strain displayed a pattern compatible with a poorly glycosylated form of AcrA with the O antigen (Figure 2A and 2B, lanes 3 and 3*). These conclusions were further supported by a immunoblot of the same samples using the α-Yersinia O:9 (Yst9-2) and the α-Brucella O antigen (M84) monoclonal antibodies (Figure 2C and 2D, respectively). These results indicated the *C. jejuni *glycosylation system was efficiently reconstituted in *Y. enterocolitica *O:9. Although the OC- mutant would be the ideal strain for the generation of the conjugate carrying the O:9 antigen, the unexpectedly low AcrA glycosylation levels in this strain prevented its utilization for the production of the conjugate. For this reason, the conjugate containing the Ye O:9 antigen was purified from the wild-type strain.

### Identification of the carbohydrates attached to AcrA by mass spectrometry

**Figure 3:**
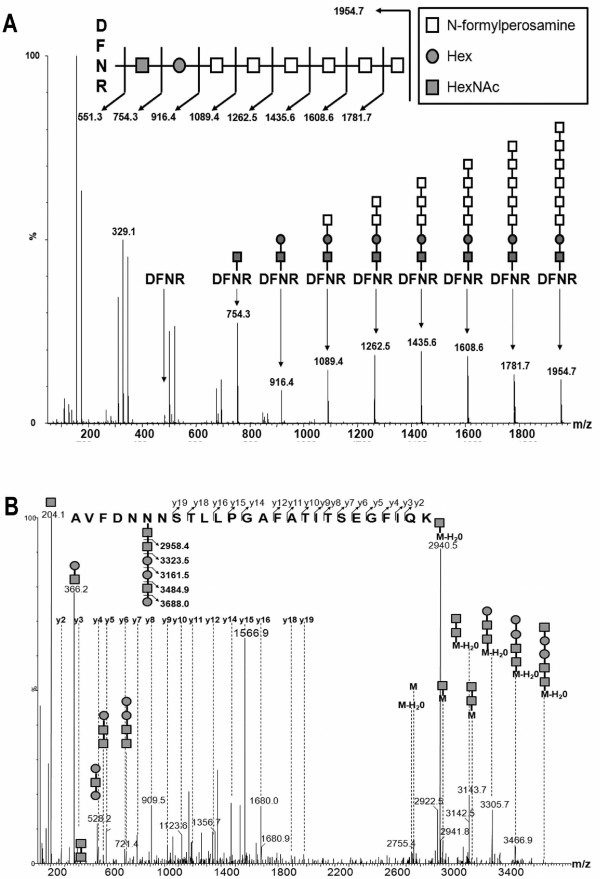
**ESI-Q-TOF MS and MS/MS analysis of of *Y. enterocolitica *O:9 glycobioconjugates**. A) MS of high molecular weight glycosylated AcrA purified from Ye O:9 WT revealed the peak 1954.7 M/Z. MS/MS of this peak showed a disaccharide of HexNAc-Hex linking a characteristic 173 M/Z pattern corresponding to the N-formylperosamine subunit to the known glycopeptide DFNR. B) MS of high molecular weight glycosylated AcrA purified from Ye O:9 O antigen mutant revealed the peak 1284.6^3+ ^M/Z. MS/MS of this peak shows the second known glycosylated site of AcrA (AVFDNNNSTLLPGAFATITSEGFIQK) modified with the hexasaccharide HexNAc-HexNAc-Hex-Hex-HexNAc-Hex.

In order to fully characterize the carbohydrates attached to AcrA, mass spectrometry techniques were employed. AcrA was purified from Ye O9 strains, separated by SDS-PAGE, the bands of interest were excised and in-gel digested with trypsin, and the resulting peptides analyzed by ESI-Q-TOF MS and MS/MS. Examination of the higher molecular weight smear from the Ye O:9 WT glycosylated AcrA by MS revealed a peak at 1954.7^1+ ^M/Z, and subsequent analysis of this peak by MS/MS identified the known glycopeptide DFNR modified with a carbohydrate moiety (Figure 3A). We identified a HexNAc-Hex disaccharide followed by a 173 M/Z repeat, which corresponds to the N-formylperosamine homopolymer, attached to the tetrapeptide DFNR, which represents one of the known glycosylation sites of AcrA (Figure 3A). MS analysis of the glycoprotein produced by O antigen deficient strain revealed a peak of 1284.6^3+ ^M/Z. MS/MS analysis of this peak identified the second known glycosylated site of AcrA (AVFDNNNSTLLPGAFATITSEGFIQK) modified with the hexasaccharide HexNAc-HexNAc-Hex-Hex-HexNAc-Hex (Figure 3B). This hexasaccharide is known as the outer core and is also present in *Y. enterocolitica *O:3. Contrary to previously published work, the outer core of Ye O:9 is therefore not structurally homologous to that of *Y. enterocolitica *O:3, despite genetic homology [28,29].

### Immune Response in BALB/c Mice vaccinated with O9-glycosylated AcrA

**Figure 4:**
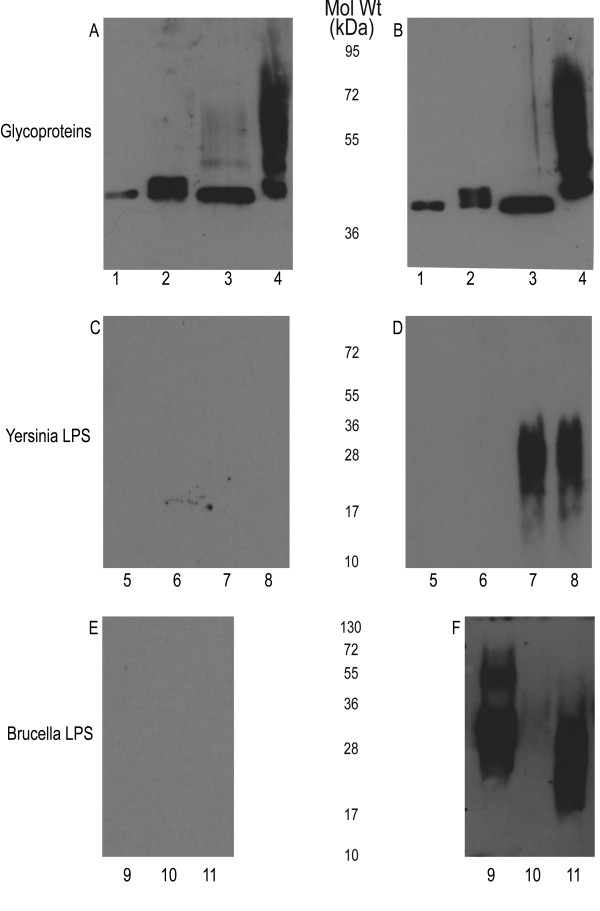
**Sera of BALB/c mice immunized with bioconjugate shows a directed IgG immune response against N-formylperosamine of *Y. enterocolitica *O:9 and *Brucella *spp. A**) Control sera and **B**) Immune sera raised by injecting purified glycoproteins containing 3 μg glycan: 1) Unglycosylated AcrA, 2) HP-, 3) OC-, 4) WT. Both sets of sera react with each glycoprotein due to the high immunostimulatory characteristic of AcrA. **C**) Control sera and **D**); immune serum (IgG response) blotted against *Y. enterocolitica *0:9 LPS from different strains from Figure 1. 5) OC-/HP-, 6) HP-, 7) OC-, 8) WT. Only the test serum was reactive against the higher molecular weight portion corresponding to the homopolymer of N-formylperosamine. **E**) Control sera and **F**) immune serum blotted against *Brucella *spp. LPSs: 9) *B. abortus*, 10) *B. melitensis*, and 11) *B. suis*. Only the immune sera are reactive against the *Brucella *LPS. Interestingly, although each LPS is comprised of N-formylperosamine, different linkages are present which may cause the difference in reactivity of the sera.

To evaluate the potential use of the glycoprotein as conjugate vaccine, the purified AcrA containing the Ye O:9 antigen was injected intraperitoneally into mice to measure the immune response as well as test subsequent protection against a challenge with *B. abortus*. The concentration of purified glycosylated AcrA was quantified as 1.77 mg/mL for protein and 0.71 mg/mL for carbohydrate, giving a protein: carbohydrate ratio of 2.48. Three separate groups of mice were injected, one with unglycosylated AcrA and two with different amounts (1.5 μg and 3 μg of carbohydrate per mouse) of glycosylated AcrA, respectively. After a second injection of glycoprotein, sera obtained from the control (Figure 4A, C, and 4E) and 3 μg group (Figure 4B, D, and 4F) were analyzed for IgG immune response by immunoblot. As expected, both sets of sera reacted strongly against AcrA (Figure 4A, b). However, when the sera were assayed against the purified *Y. enterocolitica *LPS, only the groups injected with glycosylated AcrA were reactive against purified LPS samples from *Y. enterocolitica *O:9 OC mutant and WT strains, indicating that IgG antibodies against the N-formylperosamine homopolymer were generated (Figure 4C, D). No reactivity was observed for the control sera towards the *Brucella *LPS, whereas the sera from the mice injected with glycosylated AcrA showed a strong immunoreactivity towards the *B. abortus *and *B. suis *LPS (Figure 4E, F). A very weak response was observed against the *B. melintensis *LPS.

**Figure 5:**
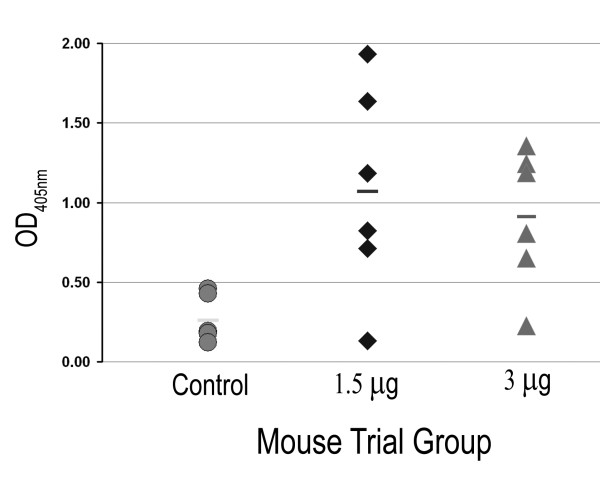
**BALB/c mice elicit an IgG immune response against *Y. enterocolitica *LPS, but is not protective against *B. abortus *infection**. **A**) ELISA response of the sera obtained from the third bleed (1/200 dilution) of the different mouse groups against *Y. enterocolitica *O:9 LPS. Microtiter plates were coated with 12.5 μg of *Y. enterocolitica *O:9 LPS. Each datum point represents the average of three replicate wells. Response was read after 1 h @ 37°C at OD_405 nm_. The bar in each set of data corresponds to the average of each group.

The generation of antibodies against the Ye O:9 antigen was further analyzed by ELISA. Each well was coated with 12.5 μg of Ye O:9 LPS (Figure 5). Of the three groups of mice, only the sera from mice belonging to the two groups inoculated with glycosylated AcrA showed an IgG response directed towards the polysaccharide. However, a high level of variation in the absorbance values was observed, with some animals showing no significant response. Interestingly, the group of mice inoculated with the lower amount of glycoprotein (1.5 μg) exhibited a higher average OD_405 nm _than the group inoculated with 3 μg. Nevertheless, the immune response was insufficient or inefficient in protecting the mice against a challenge with *B. abortus*, as no statistical difference was observed in bacterial load in the spleen of infected mice irrespective of whether they were injected with glycosylated or unglycosylated AcrA (data not shown).

### Glycoconjugates as novel antigens for the diagnosis of brucellosis

**Figure 6:**
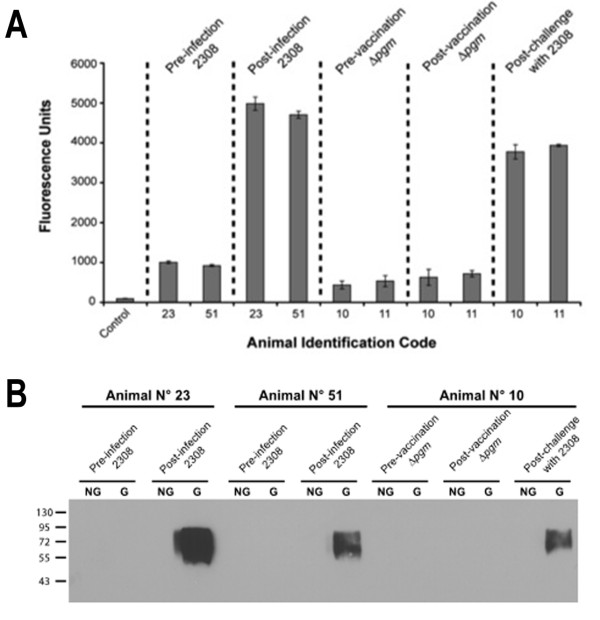
***Y. enterocolitica *O:9 bioconjugate as a promising antigen for the diagnosis of bovine brucellosis**. **A**) Magnetic bead-based immunoassay for detection of antibodies against *Brucella abortus *O-antigen. Magnetic beads coated with the AcrA-OAg glycoconjugate were incubated with the indicated bovine serum samples (dilution 1/200). Bound antibodies were detected using a Cy5-conjugated goat anti-bovine IgG. The bar graph data represents the means and standard deviation for two separate determinations. Control: magnetic beads incubated with PBS-Tween 0.1%. **B**) Immunoblot of the same bovine serum samples.

Because vaccination with the glycosylated AcrA induced the production of a specific IgG immune response against the O-antigen, we asked if this glycoconjugate could be used as an antigen for the diagnosis of the infection in cows. To test this, we immobilized AcrA (control) or AcrA-O:9 on paramagnetic microbeads (see Materials and Methods) and tested the reactivity towards sera from non-infected animals, as well as from cows vaccinated with the *B. abortus *Δ*pgm *or infected with *B. abortus *2308 strain. These animals are part of an efficacy trial to test the protective capacity of the Δ*pgm *strain [10,30] (manuscript in preparation). As mentioned earlier, Δ*pgm *is a rough strain that does not induce the production of anti-O-antigen specific immunoglobulin titers in mice. As can be observed in Figure 6A, the assay clearly differentiates non-infected from infected animals and does not react with sera from animals vaccinated with a strain that lacks a complete LPS. Additionally, it is shown that none of these sera reacted against the non-glycosylated form of AcrA in a immunoblot indicating that the IgG response detected is directed specifically towards the carbohydrate moiety of the antigen (Figure 6B). Taken together, these results strongly suggest that this novel antigen could be used for the development of new diagnostic tools for brucellosis.

## Discussion

Because the *Y. enterocolitica *O:9 and *B. abortus *LPSs share the same structure, we hypothesized that a glycoprotein carrying the Ye O:9 glycan would be able to generate antibodies recognizing *B. abortus *O polysaccharide, with the overall goal of eliciting a protective immune response against infection by this organism. We also explored if such a glycoprotein could be used for the diagnosis of brucellosis. We confirmed, using immunoblot, that mAb raised against *Yersinia *O:9 and *B. abortus *O antigens can cross-react with both structures. A glycoprotein consisting of the *Y. enterocolitica *O:9 antigen attached to the carrier protein AcrA was obtained *in vivo *by expression of the *N*-linked protein glycosylation OTase PglB of *C. jejuni *in *Y. enterocolitica *strains. The glycoprotein was purified from Ye O:9 cells and characterized by MS techniques. We confirmed the attachment of a polymer of N-formylperosamine to AcrA, which was reactive towards two mAbs directed against Ye O:9 and *B*. *abortus *O antigens, respectively. However, we also identified a previously unreported disaccharide with the formula HexNAc-Hex acting as a linker between the protein and the Ye O:9 homopolymer. A similar linker has been described in many homopolymeric O antigens in different bacterial species [31] and also in other polysaccharides such as the arabinogalactan of *M. tuberculosis *and the teichoic acids in Gram-positive bacteria ([32]. This indicates that disaccharides acting as linkers are a common feature in the synthesis of bacterial polysaccharides via the polymerase (wzy) independent pathway. Additionally, MS/MS analysis demonstrated the attachment of shorter glycan to AcrA in the wild type strain, with a structure of HexNAc-HexNAc-Hex-Hex-HexNAc-Hex. This hexasaccharide is known as the outer core and is also present in *Y. enterocolitica *O:3. Based on genetic similarities within the O:9 and the O:3 strains, it was hypothesized that the structures in both strains would be identical. However, our results demonstrate that a different structure is generated in the O:9 strain [29,33].

Injection of AcrA-O9 in mice was able to elicit an IgG immune response against the O:9 polysaccharide. Sera of inoculated mice reacted with Ye O:9 LPS, and *B. abortus *and *B. suis *O antigens. However, the reaction towards *B. melitensis *O polysaccharide was practically undetectable. *B. abortus *has both an A (α-1,2-linked homopolymer of N-formyperosamine) and an M (pentasaccharide with four α-1,2 and one α-1,3-linked polymers of the same sugar) epitope [14]. *Y. enterocolitica *O:9 antigen is comprised solely of α1,2-linked N-formylperosamine, *B. abortus *has ~98% A epitope, *B. suis *has a unique 1:7 ratio of α1,3- α1,2 linked polymer, whereas *B. melitensis *has only the M antigen of the pentasaccharide repeat [14]. These structural details help to explain why the M84 mAb against *B. abortus *does not recognize *B. melitensis *LPS, as this mAb is likely directed to an epitope absent in *B. melitensis*. However, the three *Brucella *strains reacted against the mAb α-*Yersinia *antibody, reflecting common epitopes that exist in the four structures (Figure 1B). We therefore expected that these common epitopes present in the AcrA-O9 glycoprotein would elicit antibodies that would also cross react with *B. melitensis *LPS, but the sera of the mice injected with AcrA-O9 failed to recognize B. *melitensis *LPS. This indicates that the common epitopes in all the structures are not the immunodominant ones. Interestingly, although AcrA was glycosylated with both the Ye O:9 and the OC glycan structures, only the O:9 antigen was detected by the mice sera, suggesting that the outer core is not immunogenic (Figure 4D).

A previous report suggested that a conjugate containing BSA and the O polysaccharide of *B. melitensis *was protective in mice [16]. In preliminary experiments we found that passive immunization with the Yst9 mAb was protective against *B. abortus *challenge (data not shown). These results prompted us to test the efficacy of our recombinant glycoconjugate against *B. abortus *challenge. An elevated dispersion in the titers of the vaccinated mice was obtained. However, no correlation between antibody titers and bacterial load in the spleen was found, resulting in the absence of difference in bacterial colonization of the three groups. Lack of protection could possibly be explained by the fact that *B. abortus *is an intracellular pathogen and that antibodies against this bacterium may not be able to encounter the microorganism once the infection is established. Alternatively, higher antibody titers may be necessary to elicit a protective immune response. The antibody titers were higher in the animals vaccinated with the lowest amount of glycoconjugate. It is possible that lower amounts of antigen may have to be injected to obtain protective antibody titers.

Our AcrA-O9 conjugate showed promising applications in the diagnostics of brucellosis. Diagnostics of brucellosis using lipopolysaccharide (LPS) as an antigen have been previously explored [34]. LPS are large molecules that also contain a core polysaccharide and a lipid A moiety, as well as the O antigen. LPS-based assays often suffer from false positives due to the presence of antibodies against common core antigen and lipid A, generated by other bacterial species. Here, we showed that coating magnetic beads with the AcrA-O:9 glycoprotein allows the distinction between infected and uninfected cows. The assay will be particularly useful in conjunction with vaccines like the RB51 or the Δ*pgm *strain, which do not have O antigen, as our assay will allow the distinction between vaccinated and infected animals. Further studies will be carried out to confirm the suitability, *i.e*. sensitivity and specificity of this assay for detection of bovine and human brucellosis.

Ten years have gone by since the demonstration that bacterial glycosylation systems can be successfully transferred into *E. coli*. Since then we have learned that the bacterial OTases have a relaxed specificity and are able to transfer a variety of glycans, including O antigens, to suitable protein acceptors. The experiments presented here demonstrate that an IgG immune response can be mounted against the glycan moieties in bacterial glycoproteins. Further work will expand these efforts for the generation of novel vaccines against other important bacterial pathogens. Furthermore, we also expect that the platform presented here for the detection of brucellosis will also be applied in the future for the design of additional bacterial-glycoprotein based diagnostic methods.

## Conclusions

In summary, the *C. jejuni *N-glycosylation system can be exploited to engineer designer glycoproteins. These glycoproteins can be utilized for carbohydrate characterization, vaccinology, and diagnostics. Mice injected with the YeO9-AcrA glycoconjugate were able to develop immune responses towards different *Brucella *sp., but protection was not achieved. When magnetic beads were coated with the YeO9-AcrA recombinant glycoprotein, differentiation between naïve and *B. abortus *infected bovine sera was easily discernable. These new biologically engineered glycoconjugates may be developed for a vast array of diagnostic and immunoprotective opportunities in the future.

## Materials and methods

### Bacterial strains, plasmids, and growth conditions

*Yersinia enterocolitica *O:9 strains were grown in LB media @ 37°C. Trimethoprim (100 μg/mL) and chloramphenicol (20 μg/mL) were used for plasmid selection as required. The strains and plasmids used in this study are listed in Table 1.

### Production and purification of glycosylated AcrA

Ye O:9 strains transformed with *C. jejuni *PglB (pMAF10) and AcrA (pMH5) were grown overnight at 37°C. Cultures were reinnoculated 1/100 into fresh LB media and grown at 37°C for 2.5 h (OD_600 _~0.5) and PglB_Cj _expression was induced with addition of arabinose to a final concentration of 0.2% (w/v). Four hrs after induction at 37°C, PglB_Cj _was re-induced by a second addition of arabinose to maximize glycosylation of AcrA. Cells were harvested by centrifugation after a 20 h induction period and periplasmic extracts were prepared by lysozyme treatment as described elsewhere [21]. Subsequently, the periplasmic fraction was equilibrated with 1/9 vol 10 × loading buffer (0.1 M Imidizole, 3 M NaCl, 0.2 M Tris-HCl pH 8.0) and subjected to a Ni^2+ ^affinity chromatography. The column was equilibrated with 10 column volumes of 1 × loading buffer and loaded on a HisTrap HP column (Amersham Pharmacia Biosciences) at a flow rate of 1 mL/min. The column was washed with 25 column volumes of wash buffer (0.02 M Imidazole, 0.3 M NaCl, 0.02 M Tris-HCl pH 8.0), and eluted from the column by elution buffer (0.250 M Imidazole, 0.3 M NaCl, 0.02 M Tris-HCl pH 8.0).

### LPS extraction

LPS was extracted using the hot phenol-water method as described [35]. The LPS extract was resuspended in 2 mL of distilled H_2_0.

### Sugar quantification of glycoproteins

Protocol was adapted from the total sugar quantification protocol [36]. Briefly, mix in a glass tube 90 μL ddH_2_0, 10 μL of sample, and 100 μL of 5% phenol (freshly made) in ddH_2_0. Briskly add 1 mL of conc. H_2_SO_4 _into the mixture and immediately vortex the solution for several seconds. An orange color with intensity proportional concentration will begin to develop immediately and reach a maximum after 2 h @ 30°C. Read against chemically synthesized N-formylperosamine standards @ OD_500 nm_.

### Western immunoblot

Western immunoblot was performed based on the procedure described in [37]. Samples were separated on 10% or 15% SDS-PAGE gels and transferred to a nitrocellulose membrane via semi-dry membrane transfer and analyzed with a variety of antibodies. α-AcrA antibody [38], Yst9-2 antibody [25], and Brucella antibodies M84 [39] were employed as previously described.

### Mass Spectrometry

The nickel column purified protein/glycoprotein was subsequently separated by SDS-PAGE, and the bands corresponding to the desired protein and glycoprotein were in-gel digested with trypsin (Promega) using the protocol of Shevchenko *et al*. with modification [40]. Briefly, the bands corresponding to the glycoproteins were excised and transferred to 1.5 ml Eppendorf vials. After destaining with 50 mM ammonium bicarbonate in 50% acetonitrile/water, the gel pieces were dehydrated with acetonitrile and rehydrate with 10 μl of (~2 μg) trypsin. Then the sample was left in 37°C oven for overnight digestion after addition of 50 μl ammonium bicarbonate (50 mM) aqueous. The samples were extracted using Zip-Tips (Millipore), and analyzed using a hybrid quadrupole orthogonal acceleration time-of-flight mass spectrometer (Waters, UK) equipped with ananoACQUITY Ultra Performance liquid chromatography system (Waters, Milford, MA). Briefly, 2 μl of the peptide solution was injected on to a VanGuard micro precolumn C18 cartridge that is connected to a 75 um i.d. × 150 um Atlantis dC18 column (Waters, Milford, MA). Solvent A consisted of 0.1% formic acid in water, and solvent B consisted of 0.1% formic acid in acetonitrile. After 1 min trap wash in the precolumn with solvent A at flow rate of 10 μl/min, peptides were separated using solvent gradient and electrosprayed to the mass spectrometer at a flow rate of 350 nl/min. The collision energy used to perform MS/MS was varied according to the mass and charge state of the eluting peptides. The instrument is calibrated every 1 min with GFP and LecErK using the LockSpray. For the data acquisition and analysis, MassLynx (Waters MassLynx V4.1) was used.

### Mouse trials experimental design

A group of 18 BALB/c female mice were divided into 3 groups and injected intraperitoneally with 1.5 or 3 μg of glycosylated AcrA (carbohydrate quantity) or equivalent unglycosylated control in aluminum hydroxide per mouse. The mice were injected with three doses of 2 weeks interval and the challenge was 1 week after the third dose with the virulent *B. abortus *2308 also via IP. The mice were bled via tail bleed method, resulting in a blood sample of 5-25 μL. The blood was allowed to coagulate at 24°C for 4 h, after which the samples were centrifuged for 10 min @ 13,000 rpm to isolate the blood sera. The sample was stored at -20°C until analyzed. Injections were done according to the TiterMax^® ^Kontes Pellet Pestle^® ^Homogenizer method using TiterMax^® ^Gold. A second bleeding of the mice occurred 6 weeks after the initial injection, resulting in similar sera yields. The mice were given a second injection with the same quantities of sample. The third and final bleed occurred 4 weeks after the second injection, and was also stored @ -20°C until required for analysis.

### ELISA analysis of Mouse Sera

ELISA analysis was done (Costar^® ^polystyrene High Binding plate) and optimized from (5). 100 μl of each of *Y. enterocolitica *O:9 (12.5 μg/mL LPS), *Brucella melitensis *(50 μg/mL LPS), and purified AcrA (1 μg/mL) was determined to give the optimal colorimetric response in 0.05 M sodium carbonate buffer (pH 9.8). Wells were blocked using 2.5% (wt/vol) skim milk in PBS buffer for 1.5 h. Sera from the mice were diluted 1/200 in PBS buffer, and 100 μL was placed in each well. Plates were washed 3 times with PBST. The 2^o ^antibodies conjugated to alkaline phosphatase (Biorad Laboratories) were incubated at a dilution of 1:3000 for 1 h at room temperature. Plates were washed 5 times with PBST, and were incubated with 100 μL *p*-nitrophenolphosphate substrate (1 mg/mL) in 0.05 mM sodium carbonate buffer (pH 9.8) for 1 h @ 37°C. Plates were read at OD_405 nm_.

### *Brucella *challenge against injected BALB/c mice

Groups of 6 Balb/c female mice intraperitonelly vaccinated with either 1.5 or 3 μg of the glycoconjugate were challenged 4 weeks after the second dose with 5 × 10^4 ^CFUs intraperitoneally of wild type *B. abortus *2308 and, 2 weeks post-infection, the bacterial load in the spleens determined as previously described [10].

### Magnetic-bead based immunoassays

Superparamagnectic COOH-modified microbeads (Bangs's Laboratories) were coated with the AcrA-OAg glycoconjugate in one step using EDAC [1-ethyl-3-(3-dimethyl-aminopropyl) carbodiimide hydrochloride] and NHS [N-hydroxy succinimide] reactives. Functionalized microbeads were incubated with bovine serum samples (dilution 1/200) and bound antibodies were detected using a Cy5-conjugated goat anti-bovine IgG (Biomeda). Fluorescence reading was performed using a plate fluorometer (DTX 880 Multimode Detector, Beckman Coulter).

## Competing interests

A provision patent has been filed regarding the diagnostic application of this technology.

## Authors' contributions

JAI performed genetic manipulation, protein purification, mass spectrometry analysis, and drafted the manuscript. MAF assisted with mass spectrometry analysis. AF assisted in data interpretation and experimental design. DCM performed initial work and assisted in manuscript editing. MP assisted in experimental design, initial mice testing, and editing the manuscript. CC, AEC, and DJC did experimental work regarding bacterial protection and diagnostic techniques. JEU assisted in designing mice assays, diagnostic experiments and manuscript editing. MFF designed the study, assisted with data interpretation, drafted and edited the manuscript. All authors read and approved the final manuscript.
